# Replication-Competent Oropouche Virus in Semen of Traveler Returning to Italy from Cuba, 2024

**DOI:** 10.3201/eid3012.241470

**Published:** 2024-12

**Authors:** Concetta Castilletti, Ralph Huits, Rebeca Passarelli Mantovani, Silvia Accordini, Francesca Alladio, Federico Gobbi

**Affiliations:** IRCCS Sacro Cuore Don Calabria Hospital, Negrar di Valpolicella, Italy (C. Castilletti, R. Huits, R. Passarelli Mantovani, S. Accordini, F. Alladio, F. Gobbi); University of Brescia, Brescia, Italy (F. Gobbi)

**Keywords:** Oropouche virus, bunyaviridae infections, semen, isolation and purification, arboviruses, reproductive health, vector-borne infections, PCR, viruses, Cuba, Italy

## Abstract

A febrile man in Italy who had traveled to Cuba in July 2024 was diagnosed with Oropouche fever. Reverse transcription PCR detected prolonged shedding of Oropouche virus RNA in whole blood, serum, urine, and semen. Sixteen days after symptom onset, replication-competent virus was detected in semen, suggesting risk for sexual transmission.

Oropouche virus (OROV) is an emerging zoonotic arbovirus that belongs to the Simbu serogroup of the genus *Orthobunyavirus*, family Peribunyaviridae. Natural hosts include nonhuman primates, some wild bird species, and pale-throated sloths (*1*). OROV is primarily transmitted by biting midges (e.g., *Culicoides paraensis*) and *Culex quinquefasciatus* mosquitoes. Clinical appearance of symptomatic Oropouche fever is similar to that of influenza-like illness (*1*). Self-limiting meningitis or meningo-encephalitis can occur (*1*). 

OROV has been endemic to the Amazon Region (*1*). Through September 6, 2024, a total of 9,852 confirmed Oropouche cases had been reported in Brazil, Bolivia, Colombia, Cuba, Peru, and the Dominican Republic (*2*). Travel-associated cases have been identified in Europe and the United States (*3*,*4*). In Brazil, identification of adverse pregnancy outcomes associated with OROV infection led to ongoing investigations of possible vertical transmission of the virus (*2*). In the 1980s, spontaneous abortions in pregnant women with OROV antibodies already suggested that OROV infection might be harmful during pregnancy (*5*).

On August 2, 2024, a 42-year-old man from Italy was evaluated for an acute febrile illness at the IRCCS Sacro Cuore Don Calabria Hospital, Negrar di Valpolicella, Italy. He had visited Cuba during July 19–29, 2024. The day before returning home, he experienced high fever (39.0°C), headache, and general malaise (Figure 1). After 2 days, his fever subsided, but it recurred on day 4 after symptom onset. Neurologic examination was unremarkable; no meningeal irritation was noted. The patient did not have a rash or lymphadenopathy and did not report testicular discomfort. Laboratory evaluation showed a leukocyte count within reference limits and no thrombocytopenia. Blood culture results were negative, and specific real-time reverse transcription PCR (RT-PCR) of whole blood and serum were negative for dengue, chikungunya, and Zika viruses (Appendix). We diagnosed Oropouche fever by using 2 OROV-specific RT-PCRs that both target the small (S) genomic segment (*6*,*7*), which was positive in serum, whole blood, and urine samples collected on day 4 after symptom onset. The patient recovered and was free of symptoms on day 10.

**Figure 1:**
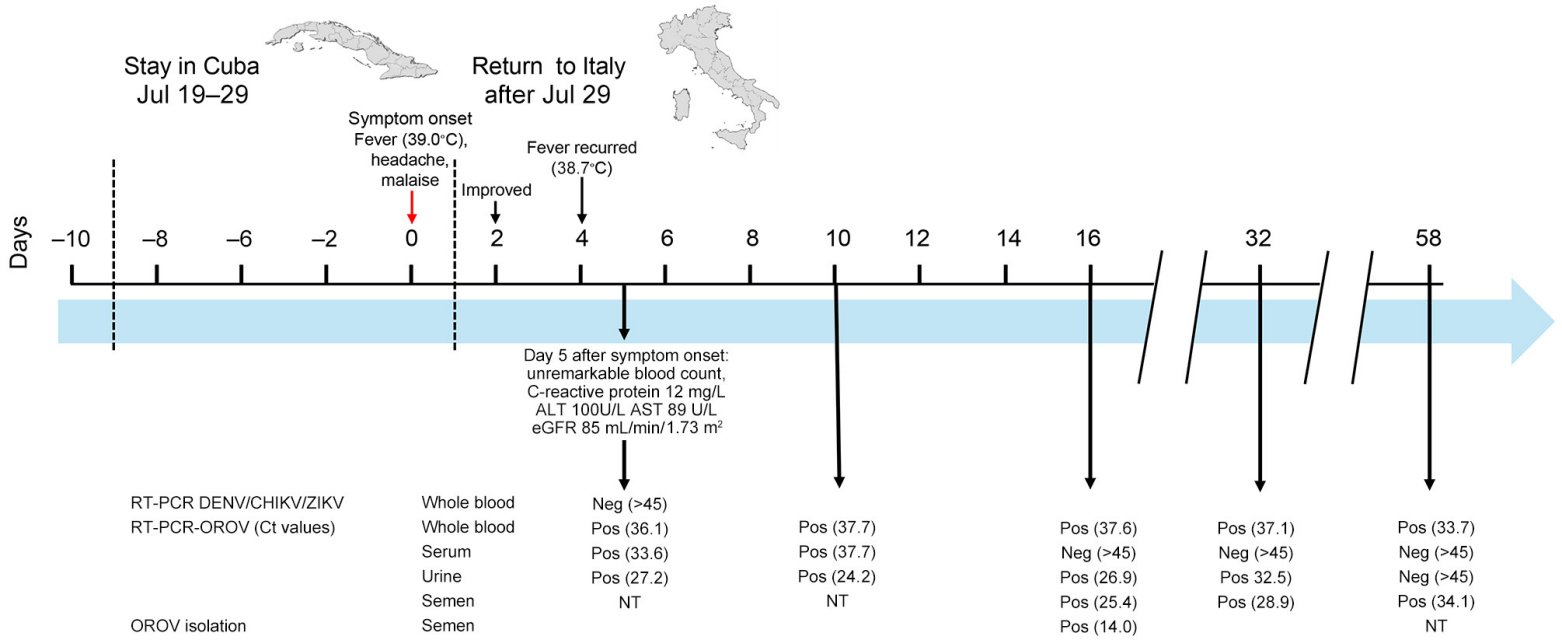
Clinical events and laboratory findings associated with OROV infection in a 42-year-old man from Italy who visited Cuba during July 19–29, 2024. Shown is clinical timeline of patient’s OROV infection, with key days indicating the onset of symptoms and the results of both endpoint and real-time RT-PCR tests of whole blood, serum, urine, and semen samples. ALT, alanine aminotransferase; AST, aspartate aminotransferase; CHIKV, chikungunya virus; Ct, cycle threshold; DENV, dengue virus; eGFR, estimated glomerular filtration rate; NT, not tested; neg, negative; OROV, Oropouche virus; pos, positive; RT-PCR, reverse transcription PCR; ZIKV, Zika virus.

RT-PCR remained positive in whole blood and urine samples obtained on days 10, 16 and 32 after symptom onset. Serum was positive for OROV RNA (cycle threshold [Ct] 37.7) on day 10 but not day 16 (Ct >45). We detected OROV RNA in fresh, unfractionated semen samples on days 16 ([Ct 25.4], 32 [Ct 28.9], and 58 [Ct 34.1]). Viral RNA levels were higher in semen than in urine or whole blood (Figure 1). On day 58, OROV was still detectable in whole blood and in semen but not in urine.

On day 16 after symptom onset, we obtained infectious OROV from semen in cell culture under Biosafety Level 3 conditions (Appendix). Virus replication was confirmed by the appearance of clear cytopathic effects (CPE) after 5 days (Figure 2) and increased OROV-RNA levels in spent cell growth medium (Ct values decreased from 25.4 to 14.0). We harvested the virus and performed subsequent passages. On day 32, we still detected shedding of OROV RNA at higher levels in semen (Ct 28.9) than in urine (Ct 32.5) and whole blood (Ct 37.1) but could no longer demonstrate replication competence.

**Figure 2:**
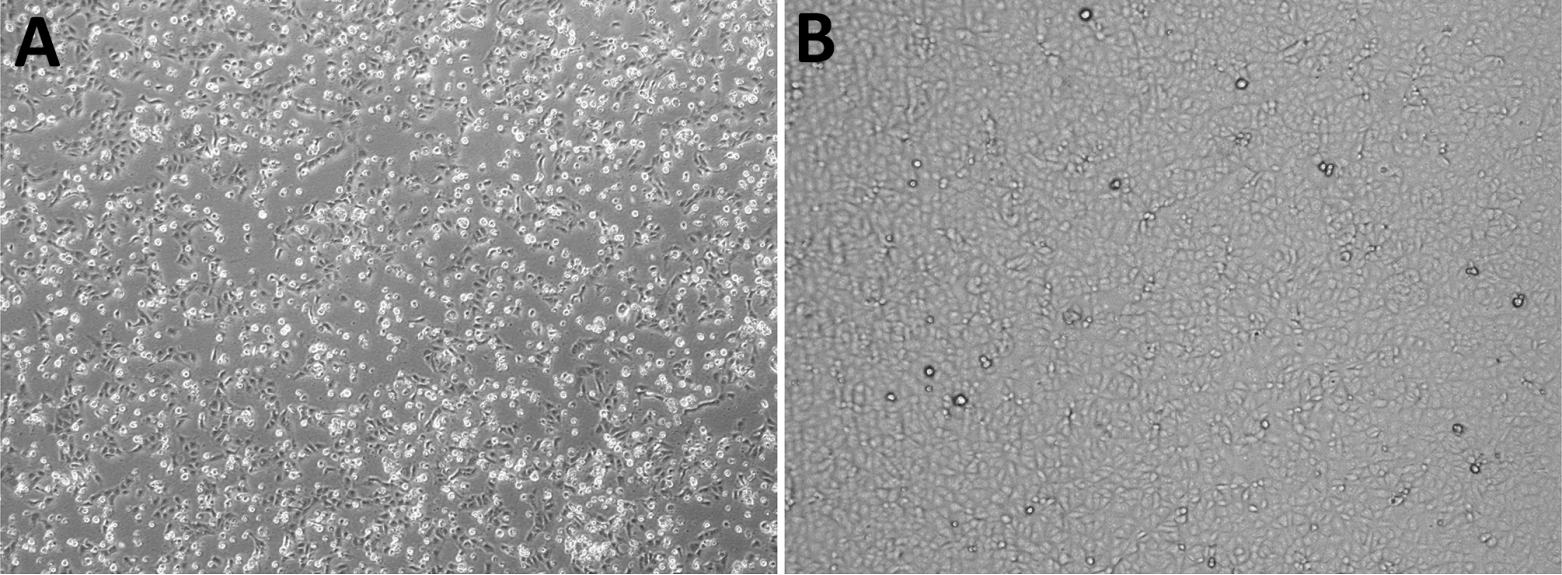
Cytopathic effect associated with Oropouche virus infection in a 42-year-old man from Italy who visited Cuba during July 19–29, 2024, observed by using light microscopy on Vero E6 cells monolayer after 5 days of incubation. A) Semen sample from patient (Appendix). B) Semen sample from uninfected control. Original magnification ×10.

Virus persistence and shedding in semen has been documented for 40 viruses from various virus families and have been most thoroughly studied for Zika and Ebola viruses (*8*,*9*). Viruses in the family Phenuiviridae, order Bunyavirales, have also been detected in human semen: Rift Valley fever virus (day 117 after symptom onset), Toscana virus (day 59), and severe fever with thrombocytopenia syndrome virus (day 30) (*8*,*9*). Orchitis and reduced fertility have been observed in association with Toscana virus infection. Infectious Schmallenberg virus has been isolated from bovine semen up to 3 months after infection (*10*), and infection in a pregnant animal can lead to severe congenital infections in the fetus.

Detectable OROV RNA in semen could result from replication in the male genital tract but also from passive diffusion of OROV. In the patient we report, blood could not be excluded as a cause for a positive RT-PCR in semen, but cross-contamination from urine seems unlikely because OROV RNA shedding persisted longer in semen than in urine.

We report prolonged shedding of Oropouche virus RNA in whole blood, serum, urine and semen and detection of replication-competent virus in 1 patient but caution against overinterpretation of our findings. Because we did not separate seminal fractions, we cannot establish an association with the cellular fraction or spermatozoa. Failure to isolate RT-PCR– detectable OROV from semen does not exclude the possibility of prolonged shedding of infectious virus.

Our findings raise concerns over the potential for person-to-person transmission of OROV via sexual encounters and may have implications for sperm banking and assisted reproductive technologies. Pending further evidence (e.g., longitudinal studies to establish the frequency and kinetics of infectious OROV shedding in semen to assess its clinical relevance), we recommend use of barrier protection when engaging in sexual intercourse if OROV is confirmed or suspected.

Appendix Additional methods for study of replication-competent Oropouche virus in semen of traveler returning from Cuba, 2024.
