# Supplementary material for: Replication-Competent Oropouche Virus in Semen of Traveler Returning to Italy from Cuba, 2024
Source: Emerg Infect Dis. 2024 Dec;30(12):2684–6. doi: 10.3201/eid3012.241470 (PMC11616654; doi:10.3201/eid3012.241470)
Supplement: Appendix — Additional methods for study of replication-competent Oropouche virus in semen of traveler returning from Cuba, 2024. [file 24-1470-Techapp-s1.pdf]

Article DOI: https://doi.org/10.3201/eid3012.241470

EID cannot ensure accessibility for supplementary materials supplied by authors. Readers who have difficulty accessing supplementary content should contact the authors for assistance.

Replication-Competent Oropouche Virus in Semen of Traveler Returning to Italy from Cuba, 2024

Appendix

Real-Time Reverse Transcription PCR (RT-PCR) Assays

We used the following specific real-time reverse transcription PCR (RT-PCR) assays from Altona Diagnostics GmbH, Germany:

- For dengue virus (DENV): RealStar Dengue RT-PCR Kit 2.0.
- For chikungunya virus (CHIKV): RealStar Chikungunya RT-PCR Kit 2.0.
- For Zika virus (ZIKV): RealStar Zika virus RT-PCR Kit 1.0.

We performed both an OROV-specific RT-PCR and an RT-PCR modified to target the S segment on whole blood, serum and urine as previously described by Lambert AJ (2009), and Naveca (2017) (*1,2*).

For the OROV RT–PCR analysis in semen, semen samples were collected into sterile cups. An aliquot 140 µL of semen was used for total RNA extraction using QIAamp® RNA viral kit.

Detection of Replication-Competent OROV in Semen

Infectious OROV was obtained from semen in cell culture under BSL-3 conditions. The seminal fluid sample (OROV Ct-value 25.4) was first diluted (1:1) with viral-inoculating-broth (VIB) 1× containing antibiotics. The mixture was kept at room temperature for 30 minutes and then diluted 1:5 in Minimum Essential Medium (MEM) and inoculated onto a Vero E6 cells semiconfluent monolayer. After adsorption (90 minutes), inoculum was removed and replaced with MEM containing 2% FBS and VIB 0.5x. Cytopathic effects were observed after 5 days by light microscopy. Viral replication was confirmed by an increased

OROV-RNA load (Ct-value 14.0) in spent cell growth medium. Subsequent passages were performed.

References

1. Lambert AJ, Lanciotti RS. Consensus amplification and novel multiplex sequencing method for S segment species identification of 47 viruses of the *Orthobunyavirus, Phlebovirus*, and *Nairovirus* genera of the family *Bunyaviridae*. J Clin Microbiol. 2009;47:2398–404. PubMed https://doi.org/10.1128/JCM.00182-09

2. Naveca FG, Nascimento VAD, Souza VC, Nunes BTD, Rodrigues DSG, Vasconcelos PFDC. Multiplexed reverse transcription real-time polymerase chain reaction for simultaneous detection of Mayaro, Oropouche, and Oropouche-like viruses. Mem Inst Oswaldo Cruz. 2017;112:510–3. PubMed https://doi.org/10.1590/0074-02760160062
